# Street-drug lethality index: A novel methodology for predicting unintentional drug overdose fatalities in population research

**DOI:** 10.1016/j.drugalcdep.2021.108637

**Published:** 2021-02-16

**Authors:** Orman E. Hall, O. Trent Hall, John L. Eadie, Julie Teater, Joe Gay, Meelee Kim, Dennis Cauchon, Rita K. Noonan

**Affiliations:** a Ohio High Intensity Drug Trafficking Area, Cleveland, OH, USA; b College of Health Sciences and Professions, Ohio University, Athens, OH, USA; c Department of Psychiatry and Behavioral Health, Ohio State University Wexner Medical Center Talbot Hall, Columbus, OH, USA; d National Emerging Threats Initiative (NETI), National High Intensity Drug Trafficking Areas (HIDTA), Norcross, GA, USA; e Institute for Behavioral Health, Heller School for Social Policy and Management, Brandeis University, Waltham, MA, USA; f Harm Reduction Ohio, Granville, OH, USA; g National Center for Injury Prevention and Control, US Centers for Disease Control and Prevention, Atlanta, GA, USA

**Keywords:** Addiction medicine, Drug users, Epidemiology, Mortality, Premature

## Abstract

**Background:**

Emerging evidence suggests the composition of local illicit drug markets varies over time and the availability and relative lethality of illicit drugs may contribute to temporal trends in overdose mortality. Law enforcement drug seizures represent a unique opportunity to sample the makeup of local drug markets. Prior research has associated shifts in the types of drugs seized and trends in unintentional drug overdose mortality. The present report builds on this work by demonstrating a novel methodology, the Street-Drug Lethality Index, which may serve as a low-lag predictor of unintentional overdose deaths.

**Methods:**

Data included administrative records of law enforcement drug seizures and unintentional drug overdose deaths in Ohio from 2009 -to- 2018. Death records and lab results from drug seizures occurring during the calendar year 2017 were transformed via the described procedure to create lethality indices for individual drugs. These indices were then summed annually to create the independent variable for a linear regression model predicting unintentional overdose deaths for all years during the study period.

**Results:**

The regression model explained 93 % of the year-to-year variance in unintentional overdose fatalities (slope = 0.009480; CI = 0.007369 to 0.011590; *t*_10_ = 10.355942; P = 0.000007; Y = 11.808982 + 0.009480X, *r*^2^ = 0.931).

**Conclusion:**

These findings contribute to a growing body of evidence that changes in the composition of the drug supply may predict trends in unintentional overdose mortality. The proposed methodology might inform future overdose prevention and response efforts as well as research.

## 1. Background

A common limitation of research assessing population-based interventions to reduce unintentional drug overdose is a failure to control for the effect of the illicit drug supply on overdose mortality. The availability and relative lethality of trafficked drugs varies geographically and over time (Drug Enforcement Administration, 2017). Changes in the composition of the illicit drug supply have been observed to parallel shifts in overdose mortality (Hall et al., 2020; Jones et al., 2018; Zoorob, 2019). Law enforcement interdiction represents a unique opportunity to assess the makeup of local illicit drug markets, with each street level confiscation representing a sampling of the drugs available at a given place and time. A recent report found an association between law enforcement seizures of illicit drugs and overdose deaths in Ohio from 2014 to 2017 (Zibbell et al., 2019). However, further research is required to show how this association might predict the incidence of unintentional drug overdose in population health research.

There is an urgent need for improved timeliness in estimates of unintentional drug overdose mortality. A significant lag often occurs between shifts in unintentional drug overdose mortality and official reports detailing these trends. Data documenting changes in unintentional drug overdose mortality are often unavailable for months, and sometimes a year or more. Yet public health officials, treatment providers, law enforcement and community groups must respond swiftly in order to save lives when shifts in unintentional overdose mortality occur. Law enforcement data, including information on the composition of seized illicit drugs, are available on a timelier basis than death certificate data in many locales. The aim of the present study is to demonstrate a novel methodology, the Street-Drug Lethality Index (SDLI), which may be employed by researchers, law enforcement and public health authorities as a low-lag predictor of the incidence of deaths due to unintentional drug overdose.

## 2. Methods

Data used in this analysis included gas chromatography-mass spectrometry (GC–MS) lab results from drug seizures conducted by Ohio Bureau of Criminal Investigation (OBCI) crime labs and overdose death records obtained from the Ohio Department of Health (ODH) for the years 2009–2018. OBCI maintains a database of drug seizures submitted by state and local law enforcement agencies to OBCI crime labs for GC–MS analysis. This database includes the type of drugs found in each seizure, the date and county of seizure, and the quantity of the drug mixture seized. Frequency of seizure was used as a proxy measure for the availability of drugs on the illicit market. Unintentional drug overdose deaths were identified using the International Classification of Diseases, Tenth Revision (ICD-10) underlying cause of death codes X40-X44. The death records of unintentional overdose decedents were reviewed for substances determined to be present at the time of death per toxicology report as well as date of death.

Death records and lab results from drug seizures occurring during the calendar year 2017 were employed via the following procedure to predict unintentional overdose deaths annually for each year 2009–2018. Calendar year 2017 was chosen as this was the peak year for unintentional overdose deaths allowing a greater number of observations to inform the calculation of our predictor variable. Table 1 presents the steps of the analysis including the calculation of sub-measures described in detail below.

Unintentional drug overdose deaths involving only one substance per toxicology report (single-drug deaths, step 1) were identified for each of the drugs listed in Table 1. Seizures in which only one drug was captured by law enforcement (single-drug seizures, step 2) were also identified. Unintentional drug overdose deaths and seizures involving more than one substance were excluded at this point of the analysis to better estimate the relative lethality of individual drugs. Lethality ratios (LR, step 3) were calculated for each drug by dividing the number of unintentional overdose deaths by the number of seizures involving individual drugs of interest in 2017.

$$Lethality\;Ratio_{Drug\;X}=\frac{Deaths\;Involving\;Only\;Drug\;X\;in\;2017}{\;Seizures\;Involving\;Only\;Drug\;X\;in\;2017}$$

SDLI (step 4) for each drug and year were determined by multiplying LR by the total number of seizures of each drug annually including single-drug seizures and mixed seizures (more than one substance present).


$$Street\;Drug\;Lethality\;Index_{Drug\;X}=\;Lethality\;Ratio_{Drug\;X}\times \;All\;Seizures\;of\;Drug\;X_{Year}$$

SDLIs of each drug were summed to produce the Summed Annual Lethality Index (SALI, step 5).

$$Summed\;Annual\;Lethality\;Index\;=\sum SDLI_{Drugs\;X,Y,Z}$$

A simple linear regression analysis was performed to predict deaths from unintentional drug overdose utilizing SALI as the independent variable and the square root transformation of observed unintentional overdose as the dependent variable. The assumption of linearity was established via scatterplot. The Durbin-Watson statistic was used to ensure independence of residuals. A plot of standardized residuals and predicted values was assessed for homoscedasticity. Normality was evaluated via histogram and Q-Q plot.

The institutional review board of Ohio University determined this study to be non-regulated. All analyses were performed in IBM SPSS Statistics for Windows (SPSS version 24; IBM Corporation, Armonk, NY).

## 3. Results

**Fig. 1.**
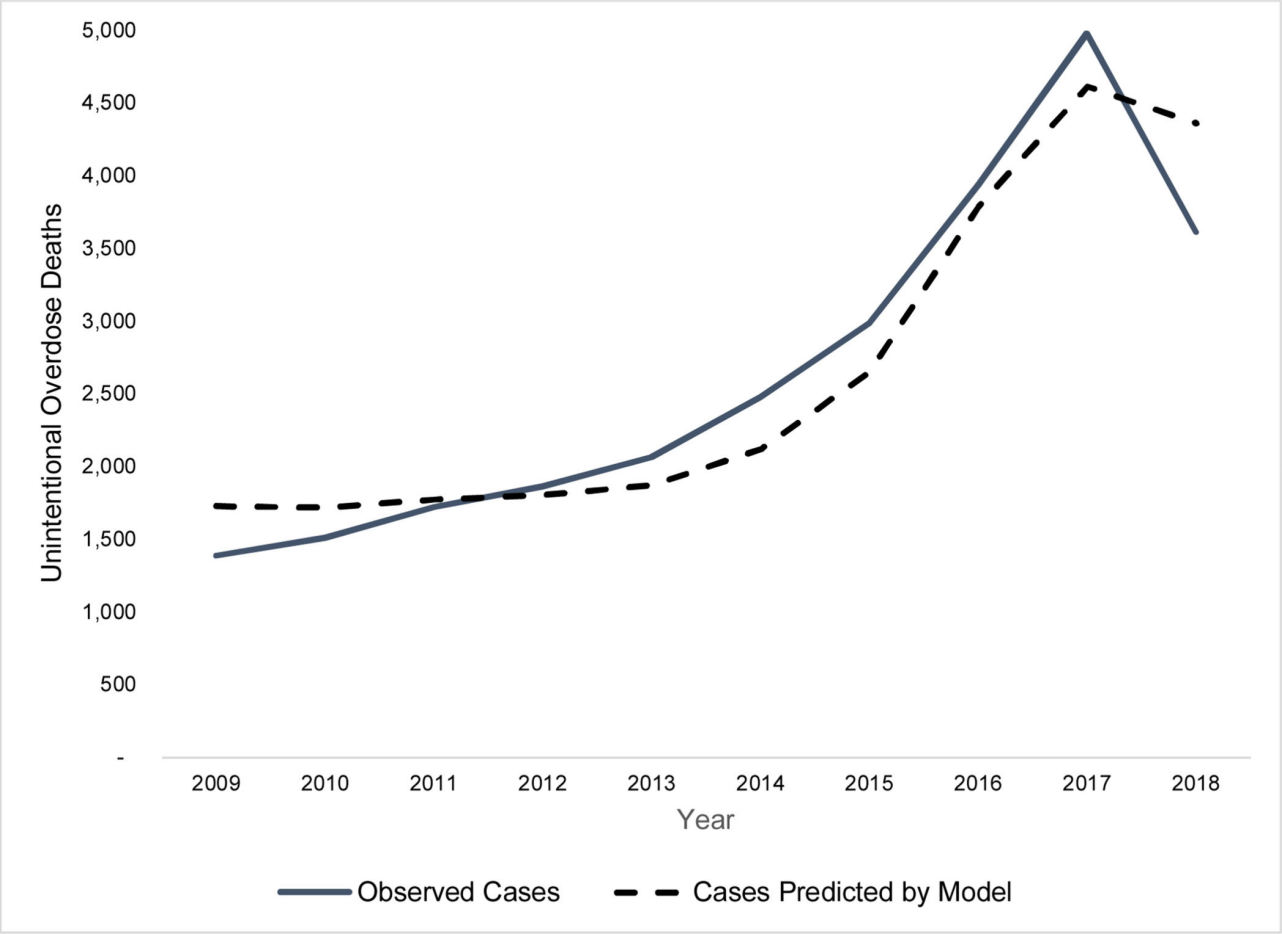
Predicted and Observed Unintentional Overdose Deaths Based on Summed Annual Lethality Index (SALI).

The Durbin-Watson statistic was 1.827 indicating independence of residuals. The assumptions of linearity and homoscedasticity were not violated per visual inspection of scatterplots. Skewness of residuals was noted via histogram and Q-Q plot. This was corrected by square root transformation of the dependent variable as noted in the methods above. The slope of the regression line was significantly greater than zero, indicating that unintentional overdose deaths increased as SALI increased. (slope = 0.009480; CI = 0.007369 to 0.011590; *t*_10_ = 10.355942; P = 0.000007; Y = 11.808982 + 0.009480X, *r*^2^ = 0.931). The model explained 93 % of the variance in unintentional overdose fatalities. Fig. 1 shows observed unintentional drug overdose deaths which occurred during the study period relative to those predicted by the model.

## 4. Discussion

The present analysis demonstrates a methodology capable of predicting the incidence of unintentional drug overdose using drug seizure and overdose mortality data, and frequency of law enforcement seizure data as a proxy measure for the local availability of lethal drugs. We have found that drug seizure data can be used to forecast unintentional drug overdose by calculating lethality indices (SDLI) for individual drugs and summing these indices (SALI) for the time period of interest. The described methodology is of high importance given the pressing need for more timely estimates of unintentional overdose mortality burden. In Ohio, public updates to the overdose death registry take up to two years, while drug seizure data reports are produced monthly. Because seizure data are available on a monthly basis in many states, trends in overdose may be predicted by the proposed novel methodology long before appearing in state death registries, empowering health systems to respond rapidly with harm reduction, primary prevention and substance use treatment services, saving lives.

Designed for ease of reproducibility by public health authorities and law enforcement officials, this methodology circumvents technical barriers to practical implementation. All necessary calculations may be performed using common desktop spreadsheet applications. Additionally, we have excluded factors such as the weight, volume or purity of seized drugs which might have slightly improved our predictions but at the cost of making SDLI a less accessible tool for public health, law enforcement agencies and researchers. Despite limiting the variables used, our model accurately predicted annual unintentional drug overdose accounting for the vast majority of year-to-year variance. Another potential limitation of our research is that it is unclear if the frequency of law enforcement seizures increases with improvements in law enforcement efforts in response to overdose trends, or if seizures and unintentional overdoses rise and fall together with the ebb and flow of lethal drugs on the illicit market. However, neither scenario negates our principle findings nor the practical utility of our approach as a low-lag predictor of unintentional drug overdose. Public health and public safety organizations should partner at all levels of government to facilitate data-sharing and predict overdose via this approach.

In addition to its anticipated public health applications, the present work has many implications for future research. Additional studies are needed to determine applications for SDLI on different time and geographic scales. Epidemiological moderation analyses may provide insight into the impact of improved treatment and prevention efforts. For example, one might determine to what extent a particular intervention moderated the relationship between SALI and observed overdose deaths (i.e. decreased mortality despite stable or increased lethality of the local drug supply). SDLI should be further investigated as a potential means of quantitatively differentiating the relative lethality of different illicit drugs, predicting unintentional overdose deaths based on drug seizures at the national level and evaluating the effectiveness of interventions designed to reduce deaths by examining disparities between observed deaths and those predicted by street-drug lethality.

## Figures and Tables

**Table 1:** Summary Statistics for Street Drug Lethality Index.

Steps 1− 3 of the Analysis by Drug			
Drug	Step 1: Pure Deaths 2017	Step 2: Pure Seizures 2017	Step 3: Lethality Ratio (LR)
Fentanyl	1497	3812	0.393
Cocaine	254	5781	0.044
Prescription Opioids	217	3650	0.059
Heroin	110	2486	0.044
Amphetamines	85	9012	0.009
Benzodiazepines	28	5437	0.005

Step 4: Street Drug Lethality Index (SDLI) by Drug and Year						
Year	Fentanyl	Cocaine	Prescription Opioids	Heroin	Amphetamines	Benzodiazepines
2009	15	252	277	134	16	16
2010	12	205	317	134	13	16
2011	11	218	323	169	17	18
2012	11	176	315	242	27	20
2013	27	202	289	291	29	19
2014	220	215	285	340	29	18
2015	565	245	314	403	40	20
2016	1435	312	298	343	61	30
2017	2123	300	270	225	98	31
2018	1989	268	258	208	131	26

Steps 5: Summed Annual Lethality Index (SALI) and Observed and Predicted Unintentional Overdose Deaths by Year				
Year	Step 5:SALI	Observed	Predicted
2009	710	1389	1730
2010	697	1511	1719
2011	756	1720	1774
2012	791	1864	1808
2013	857	2065	1872
2014	1107	2476	2123
2015	1587	2986	2652
2016	2479	3939	3788
2017	3047	4979	4617
2018	2880	3614	4362

Note: Step 1 is a count of all unintentional overdose deaths attributable to only one drug of abuse (single-drug deaths) for calendar year 2017.

Step 2 is a count of all seizure events from the Ohio Bureau of Criminal Investigation database where only one drug was present (single-drug seizures).

Step 3 is the lethality ratio calculated by dividing single-drug deaths by single-drug seizures.

Step 4 calculates the Street Drug Lethality Index (SDLI) by multiplying the total number of seizures (single-drug and mixed) by the lethality ratios calculated in step 3.

Step 5, is the summation of all annual SDLI scores, SALI. SALI is used as the predictor variable in the regression analysis.

## Abbreviations:

OBCI: Ohio Bureau of Criminal Investigation
ODH: Ohio Department of Health
ICD-10: International Classification of Diseases, Tenth Revision
LR: Lethality Ratio
SDLI: Street-Drug Lethality Index
SALI: Summed Annual Lethality Index
